# Fracture of the Neck of the Femur

**Published:** 1881-12

**Authors:** 


### FRACTURE OF THE NECK OF THE FEMUR.

(Abstract of a Paper by Prof. Wight.)

When we have admitted the uncertainty and the not infrequent impossibility of making a diagnosis of fracture of the femoral neck, we may ask the following question: *Are there any signs by which we can be reasonably sure that there is a fracture of the neck of the femur,—on the supposition that crepitus is absent, and that it is not good practice to try to find crepitus.*

In order to bring some points to bear on the settlement of this question, I have tabulated the records of twenty-one cases of fracture of the femoral neck that I have seen, some with other surgeons and some in my own practice. The records that I have tabulated contain the following measurements:

(1.) Inside measurements from the superior anterior spines of the ilium to the lower ends of the internal malleoli.

(2.) Outside measurements from the superior anterior spines of the ilium to the lower ends of the external malleoli.

(3.) Measurements from the tops of the great trochanters to the lower ends of the external malleoli.

(4.) Measurements from the bases of the tibiæ to the lower ends of the internal malleoli.

(5.) Measurements from the superior anterior spines of the ilium to a line drawn transversely in front between the tops of the great trochanters. This is the *transverse femoral line*.

The object of all these comparative measurements is to determine the possibility of original symmetry of the two limbs, and find out, as far as possible, if the injury to the hip has caused any shortening of the limb on the injured side, so that we can infer the *probability* of there being a fracture of the femoral neck.

Let me repeat, in this connection, some of the points constituting reliable surgical measurements: (1) The instrument of measurement should be an accurate steel tape-line, with feet and inches on one side, and metres and centimetres on the other side. This tape-line will not elongate under tension, *and the ordinary tape-line will elongate under tension.* (2) The measurements should be made independently; that is, when one is made, the points of the tape-line should be removed from the surgeon's hands, and new points for the other side of the body should be determined without any reference to the measurement of the first side. (3) In all ordinary cases, the leg may be measured quite accurately by semi-flexing it on the thigh, for this position brings the anterior edge of the base of the tibia markedly under the integument, so that it can be made quite as accurate a point of measurement as the superior anterior spine of the ilium. (4) In most cases the tops of the great trochanters can be so accurately compared as to make the measurements from them to the external malleoli of considerable value. (5) In all cases, the personal equation of the surgeon should be most carefully excluded from any measurement. (6) The lower limbs should be put parallel with the pelvis; for, in the ordinary measurements, adduction elongates and abduction shortens the lower limb.

(1.) In all of the twenty-one cases above recorded, there was more or less obliteration of the abdomino-femoral—or inguinal—fold on the side of the injury. This was probably due to two causes: (i.) Effusion in front of the injured femoral neck; (ii.) Contraction of the soft parts in front of the femoral neck.

(2.) About one-half of the patients were examined standing up—and when the foot of the injured side was brought down to the floor, the gluteo-femoral fold on that side was seen to be lower than the gluteo-femoral fold on the uninjured side.

(3.) There was out-rotation of the injured limb in all of the above cases. In Cases I. and II. the out-rotation was not marked.

(4.) In all the cases of impaction of the base of the femoral neck, the upper end of the femoral shaft was materially enlarged. There were probably eight such cases.

(5.) In all of the cases of impaction of the top of the femoral neck into the femoral head, the upper end of the femoral shaft was not enlarged. There were probably five such cases.

(6.) The other eight cases could not probably belong to either of the above classes.

(7.) In all of the above cases there was more or less prominence of the outside of the hip, but the gluteal region was somewhat flattened; and generally there was a fusiform enlargement of the upper part of the thigh.

(8.) In 14 of the 21 cases there was more or less asymmetry of the lower limbs. And this point is important for two reasons: First, It was determined by measuring from the tops of the great trochanters. Second, it agrees with the general fact, that about two persons out of every three, have asymmetry of the lower limbs: Hence, the above measurements from the tops of the great trochanters to the external malleoli were probably correct: *Hence, such a measurement may be recommended as a valuable aid in making a diagnosis of fracture of the neck of the femur.*

(1.) The average shortening after fracture of the neck of the femur, as shown by the inside measurement, is about 62–100 of an inch; as shown by the outside measurement, is about 55–100 of an inch; and as shown by both measurements, is about one-half inch, 58–100.

(2.) The greatest shortening was one inch and one-half—in case V.

(3.) The least shortening was zero—in case XVI. *But in that case there was an actual shortening of three-fourths of an inch.*

(4.) The average normal asymmetry of the lower limbs in the above 21 cases was 40–100 of an inch.

(5.) The average shortening of the measurement from the superior anterior spine of the ilium to the transverse femoral line was about one-half inch, again showing that the top of the trochanter major is an approximately accurate point from which to measure.

(6.) In the four following cases the injured limb appeared to be shortened one-fourth of an inch by a comparison of the inside measurements.

In no case of fracture of the femoral neck do I use force to find crepitus. I consider the other evidences of fracture—such as I have above enumerated—as sufficient to come to a practical conclusion. Nor do I give an anæsthetic in order to make an examination. In this connection let me make the following statements:

(1.) Moving the outer fragment when it is in contact with the inner fragment, will generally carry the inner fragment with it, and there will be no crepitus. And when there is impaction ordinary manipulation will not cause crepitus to be felt. Yet crepitus may at times be felt, when there is impaction of the neck of the femur.

(2.) Moving the outer fragment when it is not in contact with the inner fragment, of course will not give crepitus.

(3.) Hence, unwarrantable force will be required in order to get crepitus in many cases of fracture of the neck of the femur. And more than this—*an impacted fracture of the neck of the femur may be broken up by severe manipulation, and a patient that would have had a useful limb may be quite completely disabled for life—for an impacted fracture of the neck of the femur is the best setting of the bony fragments that the surgeon can have.*

(4.) In a suspected case of fracture of the neck of the femur, I examine all the witnesses of fracture except crepitus, and if these witnesses agree substantially, I pronounce a verdict in favor of fracture of the neck of the femur. And if there is a doubt as to the correctness of such a verdict, I give the patient the benefit of that doubt, by treating the case as if there was a fracture of the neck of the femur—and then the surgeon receives a benefit from the doubt. But if there is no fracture, the patient has had some days of needful rest, and has had a contused hip well treated.—*Proc. Med. Soc., County of Kings.*

